# Sex differences in neurobehavior and the adult hippocampal neurogenic niche: influence of traumatic brain injury and CLIP antagonism

**DOI:** 10.3389/fnbeh.2026.1768730

**Published:** 2026-02-23

**Authors:** Jaclyn Iannucci, Lavanya Venkatasamy, Michael Davis, Thao-April Nguyen, Ghazal Suhani Yadav, Victoria Nugeness, Reagan Dominy, Gabriel Maisonnave Arisi, M. Karen Newell-Rogers, Lee A. Shapiro

**Affiliations:** Department of Neuroscience and Experimental Therapeutics, Texas A&M Naresh K. Vashisht College of Medicine, Bryan, TX, United States

**Keywords:** dentate gyrus, female, fluid percussion injury, immune, male, neurogenesis, invariant chain (CD74), hippocampus

## Abstract

**Introduction:**

Traumatic brain injury (TBI) is a major cause of death and disability worldwide. Alterations to adult hippocampal neurogenesis have been identified following experimental TBI, and there are known sex differences in the response to TBI. However, few studies have investigated sex differences in neurogenesis following TBI. One of the common signatures of TBI is an inflammatory response. This includes activation of antigen presenting cells, including B lymphocytes. Previous studies have identified a pathogenic role for a B cell subset, CLIP+ B cells, following TBI. However, the role of CLIP antagonism on the adult hippocampal neurogenic niche has not been fully elucidated following a TBI, and sex differences have not been previously explored. This is extremely important because sex differences in adult neurogenesis have been previously identified. Thus, the current study was designed to test the hypothesis that CLIP antagonism after TBI would differentially influence adult neurogenesis and associated behavioral outcomes in male and female subjects.

**Methods:**

10-week-old male and female C57bl/6J mice received either lateral fluid percussion injury (FPI) or sham surgery, followed 30 min later by the administration of a CLIP antagonist peptide (CAP) or vehicle. At 35 days post-FPI, all mice underwent neurobehavioral testing using the pattern recognition test (PRT). After behavioral testing, at 60 post-FPI, harvested brains were analyzed for DCX+ newborn neurons and GFAP+ astrocytes in the hippocampus to assess the effects on the neurogenic niche.

**Results:**

FPI induced deficits in the PRT that were more pronounced in females and improved by CLIP antagonism. Immunohistological assessments revealed that female mice had reduced DCX+ neurons in the dentate gyrus and increased hippocampal GFAP+ astrocytes at 60 days post-FPI, regardless of injury or treatment condition. Further analysis showed that FPI in male mice leads to increased hypertrophy of GFAP+ radial glial in the dentate gyrus and increased presentation of hilar basal dendrites. These changes were not observed in female mice.

**Conclusion:**

The results from this study demonstrate sex differences in the neurogenic niche and associated cognitive impairment following FPI and suggest a role for CLIP after FPI in mediating these sex differences.

## 1 Introduction

Traumatic brain injury (TBI) occurs in 2–3 million Americans each year. TBI is a leading cause of death and disability (Ahmed et al., 2017; Feigin et al., 2021), and suffering a TBI is associated with an increased 30-year mortality (Elser et al., 2023). TBI is also associated with numerous post-traumatic syndromes, including depression, cognitive impairment, and an increased susceptibility to chronic degenerative disorders, such as post-traumatic epilepsy, Parkinson’s disease, and Alzheimer’s disease (Ahmed et al., 2017; Nordström and Nordström, 2018). To date, there are no effective treatments for TBI or for mitigating post-traumatic syndromes. Thus, identifying therapeutic mechanisms is of the utmost importance to improve health outcomes for the millions of people who experience a TBI each year, and the millions more living with the consequences (Elser et al., 2023).

Much like a snowflake, no two TBIs are the same. As a result, the pathophysiological response to a TBI differs in its presentation and long-term consequences. Despite this variability, there are still common behavioral and pathophysiological consequences to a TBI. This includes depression and/or cognitive impairment which are observed in ~70% and 50% of patients, respectively (Hart et al., 2012). Pathophysiological changes include inflammation and neuroinflammation (Needham et al., 2019), as well as changes to the endocrine system via alterations to the hypothalamic pituitary adrenal (HPA) axis (Hasan and Uff, 2025). A common thread that ties these behavioral and pathophysiological changes together is that they have all been demonstrated to influence adult hippocampal neurogenesis (Ziv and Schwartz, 2008; Carlson and Saatman, 2018; Gomes-Leal, 2021). Considering that altered adult hippocampal neurogenesis has been observed in multiple preclinical TBI models, examining alterations to the neurogenic niche after TBI may inform disease pathogenesis (Chirumamilla et al., 2002; Ibrahim et al., 2016; Clark et al., 2020; Correll et al., 2021; Bielefeld et al., 2024).

Adult hippocampal neurogenesis has been widely described in the dentate gyrus of a number of species, including humans and rodents (Kempermann et al., 2015), and the adult neurogenic niche has been associated with cognitive and affective function (Clelland et al., 2009; Gomes-Leal, 2021). Adult hippocampal neurogenesis is commonly altered following TBI, including the fluid percussion injury (FPI) model (Shapiro, 2017; Clark et al., 2020). These alterations include the aberrant growth, proliferation, and integration into hippocampal circuitry, resulting in impaired hippocampal function (Chirumamilla et al., 2002; Neuberger et al., 2017; Shapiro, 2017; Correll et al., 2021). Previous studies have generally identified elevated adult hippocampal neurogenesis after TBI (Villasana et al., 2015), including enhanced proliferation after TBI within 1 and 14 DPI (Dash et al., 2001; Rice et al., 2003; Sun et al., 2005; Urrea et al., 2007; Bielefeld et al., 2024). However, the more chronic effects of TBI on adult neurogenesis have not been fully characterized.

Sex differences have also been identified in the incidence and pathology of TBI in the clinic and in preclinical models, with females often showing reduced lesion size and pathogenesis when exposed to the same injury severity and location (Rubin and Lipton, 2019). Regulation of adult hippocampal neurogenesis also exhibits sex-specific effects (Barker and Galea, 2008). For example, acute stress has been found to suppress adult neurogenesis in male rats but not in females (Falconer and Galea, 2003; Hillerer et al., 2013), while cognitive tasks seem to enhance neurogenesis in males but not in females (Chow et al., 2013; Yagi et al., 2016; Yagi et al., 2020). In the context of TBI, sex differences in the neurogenic niche after injury have been identified (Neale et al., 2023), with males exhibiting more pronounced effects up to 6 weeks post-injury (Downing et al., 2025), although data on the full extent of sex differences in the neurogenic niche following TBI remain to be elucidated.

Inflammation is among the many stimuli that alter the neurogenic niche. TBI induced inflammation and neuroinflammation are initiated as part of the innate immune and neuroimmune response to injury (Wu et al., 2024). Most TBIs rapidly activate local glial and immune cells, along with the acute phase response that results in elevated levels of acute phase effector proteins (Nizamutdinov et al., 2017). Accumulating evidence also indicates that the TBI-induced innate immune response can lead to a transition to an antigen-specific adaptive immune response (Tobin et al., 2014; Daglas et al., 2019; Needham et al., 2019; Buenaventura et al., 2023). This includes activation of antigen presenting cells, including B lymphocytes (Tobin et al., 2014; Sabatino et al., 2019; Kim et al., 2021; Xiong et al., 2021; Maheshwari et al., 2023). TBI increases peripheral and infiltrating B cells (Tobin et al., 2014; Newell-Rogers et al., 2020; Newell-Rogers et al., 2022), and altered B cell signatures have been identified in the meningeal lymphatics following an FPI (Bolte et al., 2023; Iannucci et al., 2024). Therapeutically targeting the CLIP+ B cell subset is beneficial after TBI, suggesting a role in injury-induced pathogenesis (Tobin et al., 2014; Dwyer et al., 2023). CLIP, a cleavage fragment of MHC class II-associated invariant peptide (CD74), occupies the antigen binding groove of MHCII (Newell et al., 2010). CLIP antagonism after FPI was neuroprotective and anti-inflammatory (Tobin et al., 2014), and CLIP antagonism improved deficits in hippocampal neurogenesis in a mouse model of AD (Iannucci et al., 2024). Considering that TBI leads to alterations in neurogenesis and associated neurobehavioral impairment, and that targeting CD74/CLIP can improve post-injury outcomes and rescue reduced neurogenesis, the study was designed to test the hypothesis that CLIP antagonism after TBI would differentially influence the adult neurogenic niche and associated behavioral outcomes.

## 2 Methods

### 2.1 Animals

Male and female wildtype (WT) C57bl/6J (Jackson Laboratory, Bar Harbor, ME, USA; Stock #000664) (age 10 weeks) were purchased from Jackson Laboratories and allowed to acclimate for 1 week prior to experimental start. All mice were housed individually in ventilated cages in a controlled environment and maintained on a standard diet for the duration of the experiment. All work was approved by the Texas A&M Institute for Animal Care and Use Committee (IACUC; AUP #2020-0140).

Treatment groups were Sham + Vehicle (Veh), FPI + Veh, and FPI + CAP. For all groups, eight males and eight females were used.

### 2.2 Fluid percussion injury

Lateral FPI was used as a model of TBI at 12 weeks of age as previously described (Mukherjee et al., 2013; Tobin et al., 2014; Newell-Rogers et al., 2020). Briefly, mice were anesthetized with isoflurane, prepped, cleaned, and shaved. Mice were placed in a stereotaxic instrument (Stoelting, Wood Dale, IL, USA) and a 2-mm craniectomy was made over the left parietal cortex, at −1.5 mm antero-posterior and 1.2 mm medio-lateral from the bregma, making sure to keep the dura intact. The female end of a Luer-Lok syringe was then secured over the craniectomy with dental cement. Mice were then connected to the fluid percussion instrument (Custom Design & Fabrication, Model 01-B; Richmond, VA, USA) via the male Luer-Lok attachment. A 12–16 ms FPI was delivered at a pressure of ~1.2 to 1.5 atm. Sham mice received identical treatment except a pressure pulse was never delivered. After injury or sham, suture was used to close the scalp over the wound and mice were returned to their home cage resting on a heating pad. Mice were monitored to ensure they resumed normal walking, feeding, drinking, and grooming behavior.

### 2.3 Drug administration

At 30 min post sham or FPI, mice were treated with CAP as previously described (Tobin et al., 2014; Newell-Rogers et al., 2020). Mice were injected intraperitoneally (i.p.) with 1 mg/kg CAP dissolved in dimethyl sulfoxide (DMSO) and further diluted with sterile phosphate buffered saline (PBS). Veh mice received equivalent volume of DMSO dissolved in sterile PBS.

### 2.4 Pattern recognition test (PRT)

**Figure 1:**
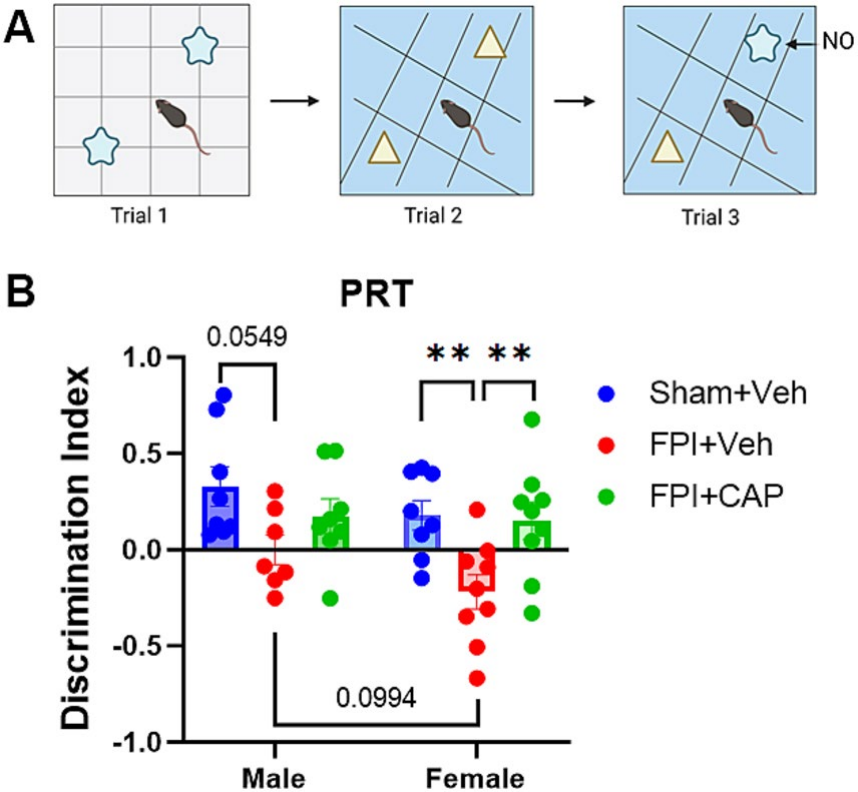
Pattern separation impairment following FPI is greater in females and improved by CAP. At 35 days post FPI, all mice underwent a pattern recognition test (PRT). **(A)** A cartoon showing the behavioral testing paradigm. In the third trial, the preference for the novel object (NO) was calculated using a discrimination index. **(B)** The deficit in pattern recognition after FPI was more pronounced in females than in males. In females CAP significantly improved pattern separation ability, whereas in males it partially mitigated the FPI-induced impairment. Data are represented as mean ± SEM; *n* = 8 per group; **p < 0.01.

The PRT (Figure 1A) was used to measure the ability of mice to recognize pattern separation, as previously described (Upadhya et al., 2016; Shetty et al., 2020; Iannucci et al., 2022). The PRT was incorporated into this study to assess a hippocampal-dependent task that is linked to intact neurogenesis (Clelland et al., 2009). The test comprised of three successive trials separated by a 1-h intertrial interval. Mice were previously acclimated to the open field test box. In the first trial, mice were placed in the open field box with a first set of two identical objects (shape 1 objects) positioned on floor pattern 1 (P1) and allowed to freely explore both objects for 5 min. In the second trial, mice were placed in the open field box with a second set of identical objects (shape 2 objects) on floor pattern 2 (P2) and again allowed to freely explore both objects for 5 min. In the third and final trial, one of the shape 2 objects from trial 2 was replaced with a shape 1 object on P2. This shape became the novel object (NO) and the shape 2 object became the familiar object (FO) on the P2 floor. Again, the mice were allowed to freely explore both objects for 5 min. Each trial was video recorded and analyzed using automated NOLDUS EthoVisionXT video tracking software (Noldus, Leesburg, VA, USA). Additional scoring was done manually by a rater blind to the condition of the mice, in which the visits to each object and time spent with each object were assessed. A discrimination index was calculated to assess preference for the NO. The discrimination index was calculated as (time spent with NO − time spent with FO)/(time spent with NO + time spent with FO).

### 2.5 Immunohistochemistry

Immunohistochemistry was used to quantify doublecortin (DCX)+ immature neurons and GFAP+ astrocytes as previously described (Shapiro et al., 2005a,b; Ribak and Shapiro, 2007; Robinson et al., 2016; Venkatasamy et al., 2021; Iannucci et al., 2022). Briefly, mice were anesthetized with Fatal Plus (Sodium Pentobarbital; 52 mg/kg, administered i.p.) and transcardially perfused with PBS through the left ventricle until the blood ran clear. This was followed by 4% paraformaldehyde (PFA) through the left ventricle. All brains were allowed to postfix in the skull for 24 h in PFA, after which they were extracted and fixed for an additional 24 h in 4% PFA. Fixed brains were cut into 44-μm thick serial sections with a freezing microtome (American Optical Corp; Model #860). For DCX, slices first underwent antigen retrieval in 1× citrate buffer at 45° for 1 h and were subsequently stained with primary DCX antibody (Santa Cruz Inc., Dallas, TX, USA) and secondary biotinylated goat anti-Rabbit IgG (Alexaflour-555; Invitrogen, Waltham, MA, USA). For anti-GFAP staining, slices were incubated mouse anti-GFAP-Cy3 conjugated antibody (1:500; Sigma-Aldrich, St. Louis, MO, USA; #C9205), free-floating, overnight at room temperature. All slices were mounted and cover-slipped with antifade reagent (Vector Laboratories, Newark, CA, USA; H-1200-10).

### 2.6 Quantification of DCX+ newborn neurons and GFAP+ astrocytes in the hippocampus

Cells were quantified in the hippocampus using unbiased stereological methods, as previously described (Robinson et al., 2016). Sections (~every 260–350 um apart) containing the dorsal hippocampus (Bregma −1.34 through −2.80) were selected for analysis. Newborn neurons were counted in the dentate gyrus, and analysis was performed for the infra- and supra-pyramidal blades of the dentate gyrus using unbiased stereology, as previously described (Iannucci et al., 2024). GFAP+ astrocytes were quantified in CA1, CA3, and the dentate gyrus hippocampal subregions in the ipsilateral hemisphere. A minimum of 3 left hippocampi were counted per animal, per antibody, within the stereological coordinates indicated above.

### 2.7 Qualitative assessment of the neurogenic niche

Previous studies have identified FPI-induced alterations to the radial glial-like astrocytes in the hippocampal dentate gyrus in the early stages after injury (Robinson et al., 2016). Thus, the current study sought to determine the distribution and morphology of radial glial cells and their processes at a more chronic post-FPI timepoint. To determine the extent of GFAP+ radial glial processes in the upper and lower granule cell layer (GCL), images were rated on a scale of 1–3 by a blinded rater. A score of 1 indicates little to no GFAP+ radial glial processes coursing through the GCL. A score of 2 indicates a moderate number of processes, and a 3 indicates that many radial glial processes were observed in the GCL. In addition, the number of GFAP+ radial glia cell bodies was assessed in the subgranular zone (SGZ) using a similar scale of 1–3, as determined by a blinded rater. Finally, because previous studies have shown altered morphology of the radial glial-like cells at the base of the GCL, this study examined the morphology of these cells using a scale of 1–3. In this case, a 1 indicating smaller more compact cell bodies, a 2 indicating moderate cell body hypertrophy, and a 3 indicating extensive hypertrophy. All scoring of GFAP+ radial glia was performed by a single blinded rater. A minimum of 2 hippocampal slices were used per mouse, and scores for individual slices were averaged for each mouse. The hippocampal coordinates were matched across all groups (Bregma −1.34 through −2.80).

For assessment of hilar basal dendrites, a similar qualitative method was used with images rated on a scale of 1–3. A score of 1 indicates no hilar basal dendrites. A score of 2 indicates some hilar basal dendrites. A score of 3 indicates many hilar basal dendrites. All hilar basal dendrites were scored by a single blinded rater. A minimum of 2 hippocampal slices were used per animal, and scores for slices were averaged within each mouse. The hippocampal coordinates were matched across all groups (Bregma −1.34 through −2.80).

### 2.8 Statistical analysis

Statistical analysis was carried out using both GraphPad Prism (GraphPad Software, Boston, MA, USA; Version 9.0). Behavior and immunohistochemical data were analyzed by one-way and two-way analysis of variance (ANOVA) with post-hoc multiple comparisons done using the Holm-Sidak correction for planned comparisons. This study was designed *a priori* to include sufficient n to enable detection of both main effects and interaction effects between sex and treatment. For all statistical testing, significance was considered *p* ≤ 0.05 and a trend was considered at *p* = 0.0501–0.1.

## 3 Results

### 3.1 Female mice exhibit greater pattern recognition impairment after FPI that is ameliorated by CAP treatment

The PRT is used to assess pattern separation ability, a cognitive behavioral test that has been linked with adult hippocampal neurogenesis (Clelland et al., 2009; Creer et al., 2010; França et al., 2017). At 35 DPI, male mice exhibited a modest trend toward impairment in the PRT (*p* = 0.0549, NS), and female mice were significantly (*p* < 0.01) impaired in the PRT compared to Sham + Veh. The more pronounced deficit in female FPI + Veh mice included a slight trend toward a reduction compared to male FPI + Veh (*p* = 0.0994, NS). In both male and female mice, CAP treatment ameliorated the FPI-induced impairment in the PRT, although this improvement was more robust in female mice (*p* < 0.01 vs. FPI + Veh; Figure 1B), and possibly attributable to the greater deficit in females.

### 3.2 Sex differences and injury effect in the number and morphology of DCX+ immature neurons in the dentate gyrus

**Figure 2:**
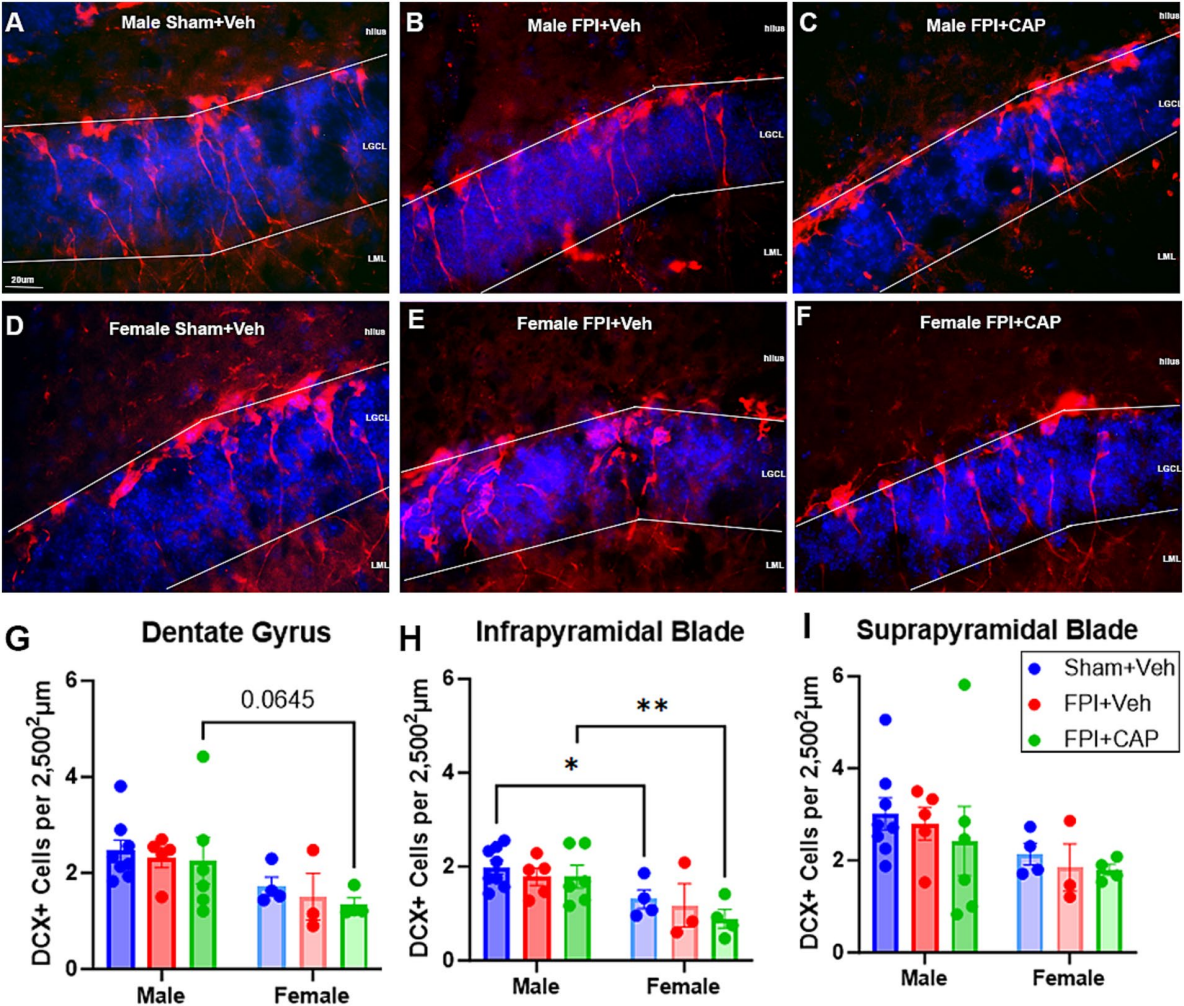
Female mice have fewer DCX+ immature neurons than males. **(A–F)** Fluorescent micrographs of the infrapyramidal (lower) blade of the dentate gyrus (DG) are shown. Although there was no injury effect at 60 DPI **(G–I)**, female Sham + Veh mice had significantly less DCX+ cells than males in the infrapyramidal blade, as did female FPI + CAP mice compared to male FPI + CAP mice **(H)**. Data are represented as mean ± SEM, *n* = 4–8 per group. Scale bar in **(A)** = 20 μm for **(A–F)**. *p < 0.05, **p < 0.01.

Pattern separation ability has been linked to hippocampal neurogenesis (Clelland et al., 2009) and impaired hippocampal neurogenesis has been previously identified following FPI (Shapiro, 2017). Thus, in the current study, a systematic and unbiased quantification of DCX+ immature neurons (Figures 2A–F) was carried out in the dentate gyrus at 60 DPI. The results show that the number of DCX+ neurons is lower in female mice, regardless of treatment group. Two-way ANOVA revealed a significant main effect for sex (*F*(1,24) = 8.590, *p* < 0.01) in the total dentate gyrus, highlighted by a trending reduction in female FPI + CAP mice compared to male FPI + CAP mice (*p* = 0.0645, NS; Figure 2G). This reduction in females was most pronounced in the infrapyramidal blade of the dentate gyrus, where two-way ANOVA revealed a significant main effect of sex (*F*(1,24) = 14.88, *p* < 0.001). This included significantly reduced DCX+ cells in female Sham + Veh (*p* < 0.05 vs. male Sham + Veh) and female FPI + CAP (*p* < 0.01 vs. male FPI + CAP; Figure 2H). There were no significant differences found in the suprapyramidal blade (Figure 2I).

**Figure 3:**
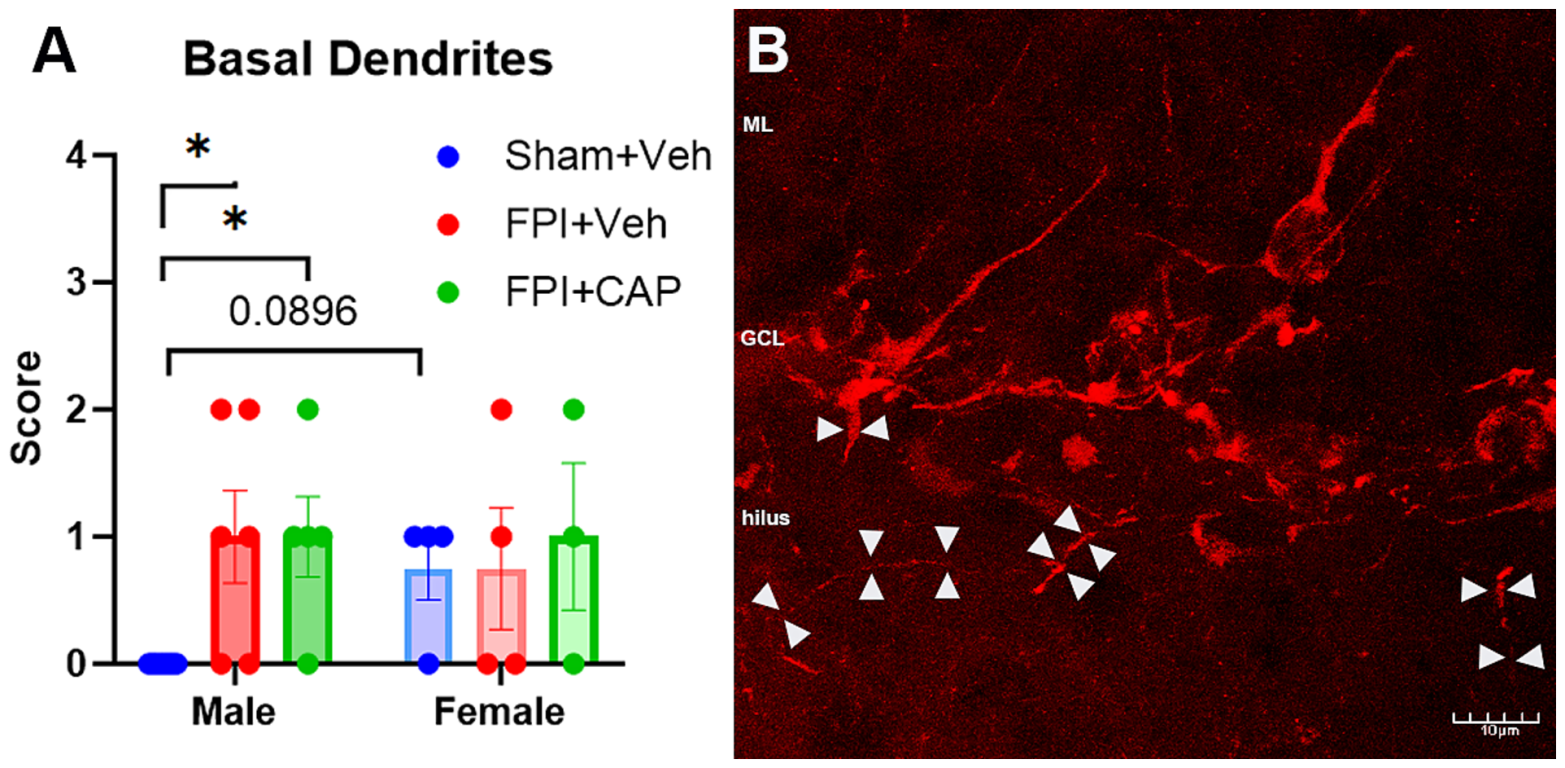
Hilar basal dendrites in the dentate gyrus at 60 DPI. **(A)** Qualitative assessment of the presence of hilar basal dendrites revealed a significant increase in male FPI + Veh mice, and in male FPI + CAP mice compared to male Sham + Veh mice. No injury effects on the basal dendrites were observed in the female mice. Interestingly, female sham mice exhibit a slight trend toward a greater number of basal dendrites than male sham mice. **(B)** A confocal micrograph depicting a male FPI + Veh mouse to show the appearance of DCX+ hilar basal dendrites extending from the cell body and coursing deep into the hilus (white arrowheads). Data are represented as mean ± SEM, *n* = 6–8 per group. Scale bar in **(B)** = 10 μm. *p < 0.05.

While there were not FPI-related effects in the number of DCX+ cells, there were sex differences in the effect of FPI on basal dendrites in the dentate gyrus. Assessment of the presence of hilar basal dendrites revealed an increase in basal dendrites in male FPI mice compared to sham (*p* < 0.05) at 60 DPI. This increase was not observed in females and was not improved by CAP treatment (Figure 3). These findings suggest chronic aberrant neurogenesis that is greater in male mice after FPI.

### 3.3 Region specific response of GFAP+ astrocytes in the hippocampus of male and female mice after FPI

**Figure 4:**
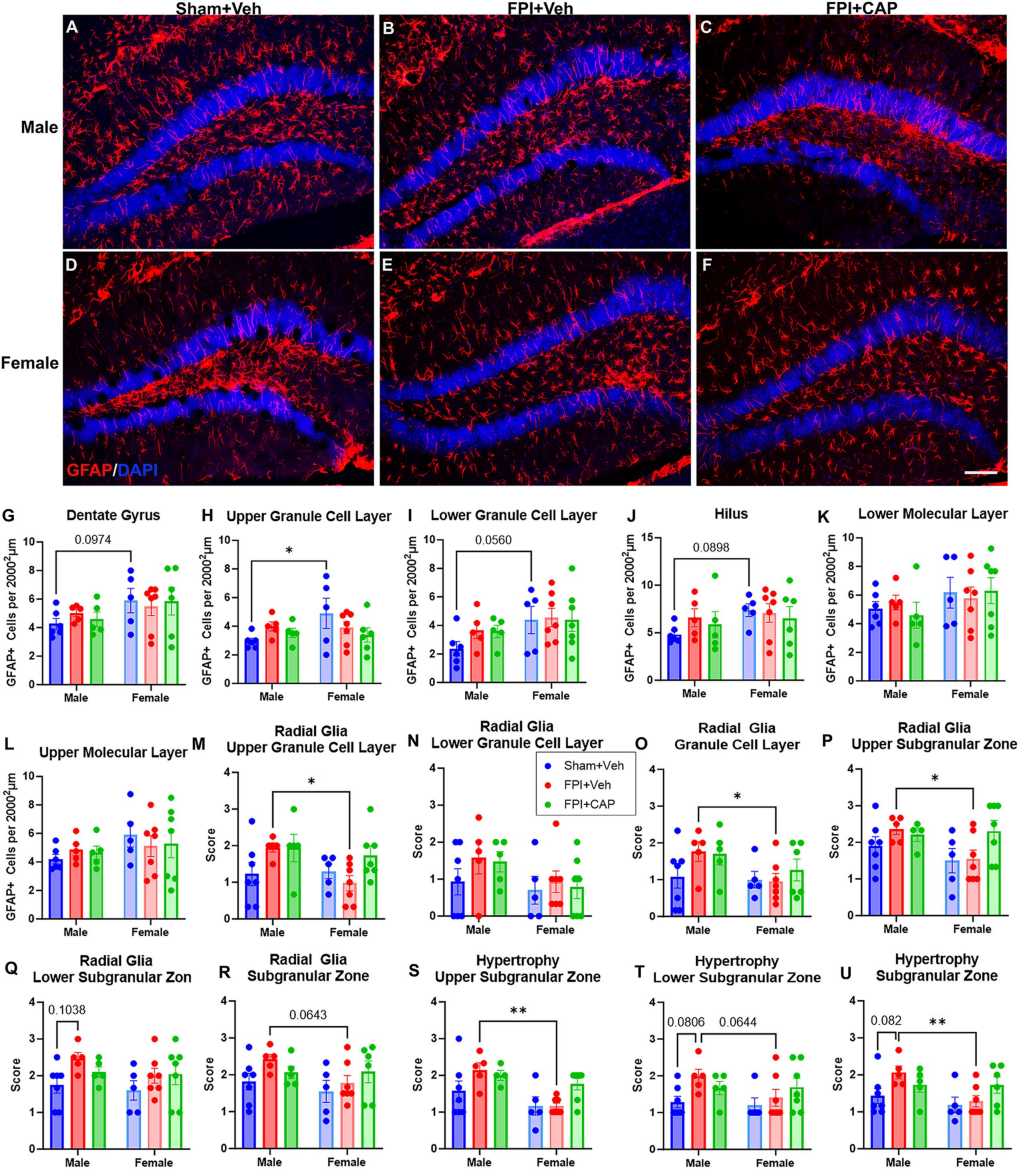
Greater numbers of GFAP+ astrocytes are seen in the dentate gyrus (DG) of female mice. **(A–F)** Fluorescent micrographs are shown to illustrate the typical appearance of GFAP+ astrocytes in each of the experimental groups. As can be seen in these images, the female sham mice have more GFAP+ cells in the DG than the male sham mice **(G)**. In the sublayer specific analyses **(H–L)**, significant increases and trends toward increased GFAP+ cells in the female sham mice were also observed in the upper **(H)** and lower **(I)** granule cell layers and the hilus **(J)**. Conversely, following FPI, male FPI mice have significantly more GFAP+ radial glial processes than females in the upper **(M)** and total **(O)** granule cell layers, and more GFAP+ radial glial cells in the upper **(P)** and total **(R)** subgranular zones. These GFAP+ cells in the subgranular zone of the male FPI + Veh mice also appear significantly hypertrophied compared to the female FPI + Veh mice **(S–U)**. Thus, in sham mice, the female mice have greater numbers of GFAP+ astrocytes in the dentate gyrus, but at 60 DPI, male mice have greater numbers of, and larger GFAP+ cells in the dentate gyrus, as compared to female mice at 60 DPI. Data are represented as mean ± SEM, *n* = 6–8 per group. Scale bar in ***F*** = 250 μm for all images. ^*^p < 0.05, ^**^p < 0.01.

Antagonism of CD74-associated signaling after FPI has previously been shown to modulate the acute astrocytic response to injury (Newell-Rogers et al., 2020). Here, GFAP+ astrocytes (Figures 4A–F) were quantified in the hippocampal subregions to determine the chronic effects of FPI and CAP antagonism in male and female mice.

In the dentate gyrus there is an overall increase in GFAP+ cells in female mice, regardless of treatment group. Two-way ANOVA revealed a strong trend in the main effect for sex (*F*(1,28) = 4.182, *p* = 0.0504, NS) in the total dentate gyrus. The analysis also shows a modest increase in female Sham + Veh compared to male Sham + Veh in the overall dentate gyrus (*p* = 0.0974, NS), the hilus (*p* = 0.0898, NS), and the lower GCL (*p* = 0.0560, NS). A significant increase was observed in the upper GCL of female Sham + Veh mice (*p* < 0.05) compared to male Sham + Veh mice (Figures 4G–J), and no differences were observed in the lower (Figure 4K) and upper (Figure 4L) molecular layers.

In the rodent dentate gyrus, GFAP+ radial glial-like cells are one type of neuronal precursor. The newborn granule cells are one of the several different types of offspring these radial glial-like astrocytes can produce (Kriegstein and Alvarez-Buylla, 2009). The radial glial-like astrocyte mothers also provide a scaffold for the growth, migration, and integration of the newborn neurons into the granule cell layer (Shapiro et al., 2005a; Shapiro et al., 2005b; Ribak and Shapiro, 2007). However, following TBI, these radial glial-like astrocytes undergo morphological transformation into an ectopic glial scaffold (Shapiro et al., 2005a,b; Shapiro and Ribak, 2005; Robinson et al., 2016), which provides an anatomical substrate for the aberrant growth and integration of newborn neurons (Shapiro et al., 2005a; Shapiro et al., 2005b; Ribak and Shapiro, 2007). Given the FPI-associated alterations to basal dendrites identified above, the current study examined the alterations to the GFAP+ radial-glial like cells at the base of the dentate gyrus GCL at 60 DPI. Qualitative assessment revealed a sex difference in the effect of FPI on the presence of radial glial processes in the GCL. There were significantly more processes in male FPI + Veh mice compared to females in the upper GCL and total GCL (*p* < 0.05, *p* < 0.05 respectively). No sex differences were identified in the sham or CAP-treated mice (Figures 4M–O). Assessment of the number of GFAP+ radial glial cells in the SGZ indicated a significant increase in male FPI + Veh compared to females in the upper SGZ (*p* < 0.05) and a trend in the total SGZ (*p* = 0.0643, NS). This effect was not identified in the CAP-treated mice (Figures 4P–R). Morphological assessment of GFAP+ cells in the SGZ revealed significantly more hypertrophy in male FPI + Veh compared to female FPI + Veh. This includes significant increases in the upper SGZ (*p* < 0.01) and total SGZ (*p* < 0.01), and a trend in the lower SGZ (*p* = 0.0644, NS; Figures 4S–U).

**Figure 5:**
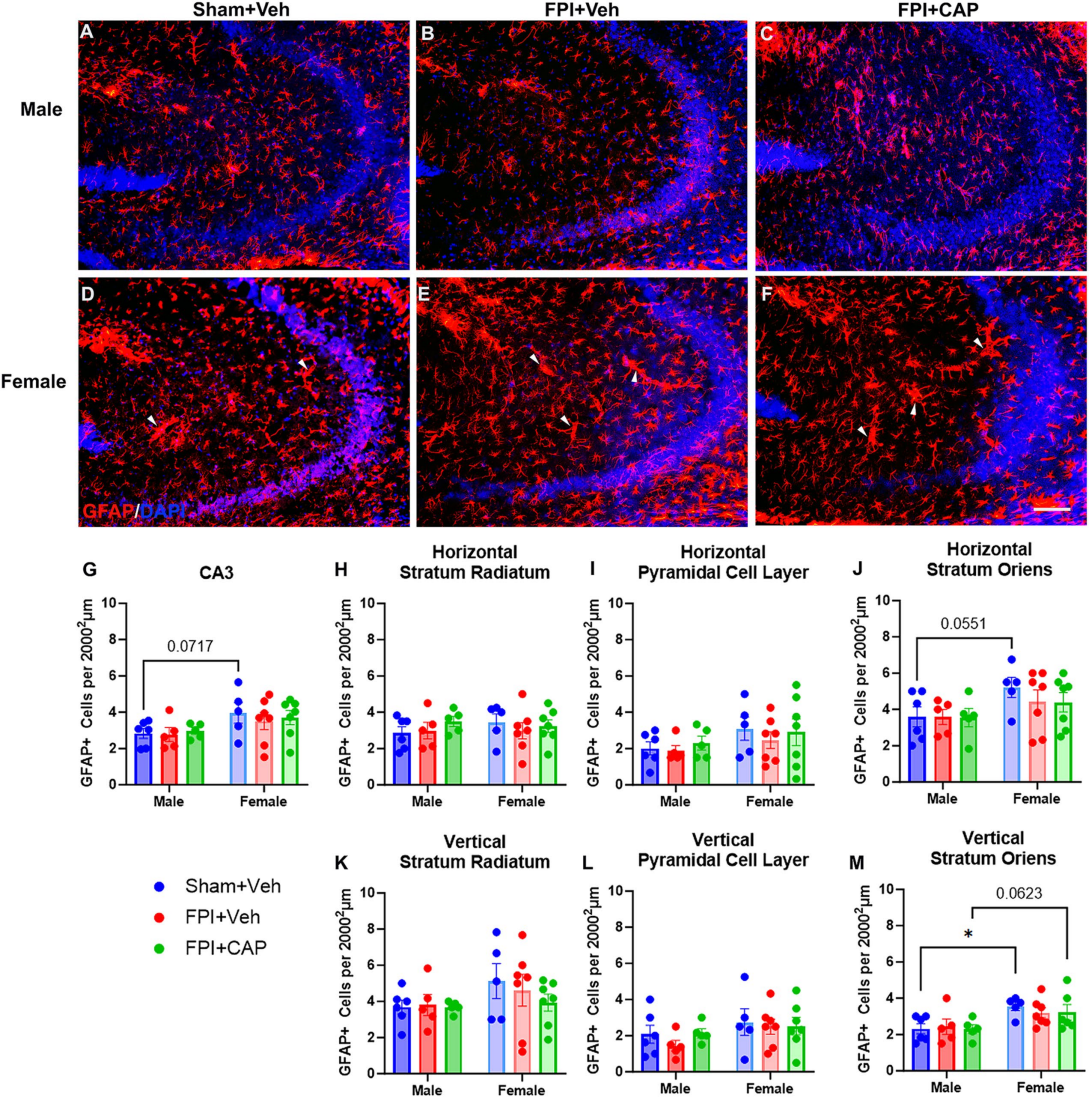
Female mice exhibit increased GFAP+ astrocytes in CA3. **(A–F)** Fluorescent micrographs showing GFAP+ astrocytes in CA3. For the purposes of our stereological analysis, the portion of CA3 that curves is considered the ‘vertical’ portion, whereas the ‘horizontal’ portion is that which extends toward the hilus. As can be seen in the combined **(G)** and layer specific **(H–M)** analyses of these regions, the total CA3 **(G)**, as well as the horizontal **(J)** and vertical **(M)** portion of stratum oriens exhibit significant or strong trends toward a greater number of GFAP+ astrocytes in female sham mice compared to male sham mice. In the vertical portion of stratum oriens, also note the trend toward a greater number of GFAP+ astrocytes in female FPI + CAP compared to male FPI + CAP mice. It is pertinent to note that although the size of astrocytes was not quantified in CA3, female mice consistently exhibit some impressively large astrocytes of unknown significance (white arrows in **D–F**). Data are represented as mean ± SEM, *n* = 6–8 per group. Scale bar in ***F*** = 250 μm for all images. *p < 0.05.

Similar to the dentate gyrus, analysis of CA3 Figures 5A–M revealed a significant main effect of sex (*F*(1,29) = 6.527, *p* < 0.05), with female mice exhibiting increased GFAP+ cells compared to males, including a trending increase in the Sham + Veh group (*p* = 0.0717, NS; Figure 5A–M). CA3 sublayer specific sex differences indicate that female Sham + Veh have a greater GFAP+ astrocytic presence in the horizontal and vertical portions of stratum oriens (*p* = 0.0551, NS; *p* < 0.05, respectively), as compared to male Sham + Veh (Figures 5J,M). There was also a trending increase in the vertical stratum oriens in the female FPI + CAP group compared to their male counterparts (*p* = 0.0623, NS; Figure 5M). No significant differences were identified in CA1, including in all three sublayers (stratum radiatum, pyramidal cell layer, and stratum oriens; Supplementary Figure 1).

## 4 Discussion

The current study identified sex differences in cognition and the adult hippocampal neurogenic niche following FPI. The current study also showed that antagonizing CLIP ameliorated some of the FPI-induced changes. Pathophysiological differences were observed in the morphology of the immature neurons and in the hippocampal astrocytes. Targeting CLIP improved the cognitive impairment in both male and female mice and selectively influenced different aspects of the neurogenic niche.

Clinically, TBI frequently induces cognitive impairment (Arciniegas et al., 2002; Kinnunen et al., 2010; Skandsen et al., 2010; Godbolt et al., 2014; McInnes et al., 2017; Bryant et al., 2023). Among the strengths of the FPI model is that it induces a consistent injury that includes various types of cognitive impairment (Lifshitz et al., 2007; Carlson et al., 2017; Yasmin et al., 2022). Previous studies have shown that antagonizing CLIP after FPI was neuroprotective, but it did not influence the peri-injury astrocytes in male mice (Tobin et al., 2014; Newell-Rogers et al., 2020). However, these previous studies did not examine hippocampal astrocytes, functional neurobehavioral outcomes, or sex differences. The current study expands on these previous findings by showing that CLIP antagonism improved the FPI-induced impairment in PRT at 35 DPI, and this improvement was more pronounced in female mice. The current study also showed that the injury-induced PRT impairment is greater in female FPI mice than in males. It is notable that pattern recognition performance has been linked to hippocampal neurogenesis (Clelland et al., 2009; Creer et al., 2010; França et al., 2017), and the impairments in PRT and neurogenesis were greater in the female mice.

Few studies have investigated sex differences in the effect of TBI on hippocampal neurogenesis. In a rat model of repetitive mild closed head injury, the acute increase in cell proliferation in the dentate gyrus was more pronounced in males (Neale et al., 2023). This is consistent with the data from the current study in which the female mice exhibit fewer immature neurons at 60 DPI, suggesting the possibility of increased proliferation in the male mice after FPI. Conversely, a different study using the controlled cortical impact (CCI) model of TBI, showed the loss of newborn neurons and impaired dendritic development was more pronounced in males than females at 6 weeks post-injury (Downing et al., 2025). It is pertinent to note that the CCI model induces direct, physical deformation of the hippocampus. Thus, the mechanisms by which CCI influences altered neurogenesis may be vastly different than for more mild injuries such as FPI, in which the lesion only impacts the most superficial portions of neocortex.

While there were no differences in the number of DCX+ cells identified at 60 DPI, the reduced number of DCX+ cells in female mice was most notable in the FPI + CAP group, suggesting that CAP treatment may selectively influence neurogenesis in female mice. Although previous studies have shown that CAP increased neurogenesis in a mouse model of Alzheimer’s disease, this was in male mice (Iannucci et al., 2024). While future studies are needed to more fully elucidate the distinct differences in the influence of CAP on neurogenesis in male and female mice, it is possible that CAP influences neurogenesis via its anti-inflammatory actions (Newell et al., 2010; Van Beusecum et al., 2019). Inflammation has been shown to be detrimental to hippocampal neurogenesis (Ekdahl et al., 2003), in a sex specific manner (Chandwani et al., 2023; Lee et al., 2025). Therefore, it is possible that CAP differentially influences inflammation in male and female mice, resulting in different influences in neurogenesis.

It is pertinent to note that in the current study the estrous cycle was not monitored. This is important because it is possible that in female mice, CAP may be interacting with sex hormones to produce a greater reduction DCX+ cells. As hormonal variability may influence various aspects of the neurogenic niche, including changes to progesterone and estradiol (Tanapat et al., 1999; Barker and Galea, 2008; Pawluski et al., 2009; Duarte-Guterman et al., 2015; Mirzaeian et al., 2024), it is possible that the results of the current study would differ if injury or analyses were performed during a specific phase of the estrous cycle. It is also possible that CAP may differentially influence female mice at different phases of the estrous cycle, including neuronal stem cell proliferation, differentiation, integration, and survival (Woolley and McEwen, 1992; Tanapat et al., 1999; Rummel et al., 2010; Tzeng et al., 2014; Mahmoud et al., 2016; Mirzaeian et al., 2024). Future studies are needed to more fully investigate these possibilities.

Adult hippocampal neurogenesis is intimately associated with the radial glial-like astrocytes at the border between the SGZ and GCL (Shapiro et al., 2005a,b; Ribak and Shapiro, 2007). Few studies have examined sex differences in this population of cells following TBI. In the current study, these astrocytes in male mice exhibit greater numbers and hypertrophy at 60 DPI than in female mice. Previous studies have shown that hypertrophied astrocytes provide a scaffold for the ectopic growth and migration of the immature neurons (Shapiro et al., 2005a,b). Examination of the DCX+ ectopic immature neurons in the hilus did not reveal any differences at this time point (not shown). However, there were differences observed in the DCX+ hilar basal dendrites along the ectopic glial scaffold formed by the hypertrophied radial glial-like astrocytes, with males exhibiting increased growth of hilar basal dendrites after FPI. There were no differences in the radial glia observed in male FPI + CAP mice compared to Sham + Veh, suggesting that CAP ameliorates the FPI-induced formation of the ectopic glial scaffold, although no sex differences were identified in the CAP-treated groups. Thus, CAP may have normalized the sex differences in radial glial following injury. How this relates to neurogenesis and neurobehavioral improvements remains to be elucidated.

In addition to the radial glial-like astrocytes, analysis of GFAP+ astrocytes in the other regions of the hippocampus revealed a greater number of astrocytes in female mice, regardless of treatment group. The increased number of astrocytes were most notably in CA3 and the dentate gyrus. Previous studies have identified sex differences in astrocytes following TBI, although the studies are somewhat conflicting (Muñoz-Ballester and Robel, 2023). One study found that females exhibit increased GFAP expression after a CCI (Jullienne et al., 2018), including hypertrophic GFAP+ astrocytes at 2 and 8 days post-injury, and increased GFAP in the perilesional cortex at 30 days post-injury (Rauk et al., 2025). Another study found a significant increase in GFAP+ astrocytes in males compared to females in the cortex, dentate gyrus, and thalamus in the first 7 days post CCI, but this difference was not found at 30 days post-injury (Villapol et al., 2017). It is likely that differences in injury types and DPI greatly influenced the variability across studies. Furthermore, in non-injury conditions, astrocytes exhibit sex differences in morphology and activation (Gozlan et al., 2024), with females expressing more hippocampal astrocytes with smaller processes (Mouton et al., 2002). Therefore, the sex differences identified in the current study at 60 days post-FPI add to these previous findings.

In conclusion, these findings highlight key changes to neurogenesis-associated behavior and the neurogenic niche after injury that differ by sex. This includes PRT deficits following FPI that are more pronounced in females and improved by CAP. This study also demonstrates chronic sex differences in the neurogenic niche in FPI that can be partially mitigated by CLIP antagonism. Future investigations are needed to more fully probe the mechanisms responsible for these sex differences to the adult hippocampal neurogenic niche and the associated neurobehavioral outcomes.

## Data Availability

The data sets presented in this article are not readily available due to ongoing analyses. Request to access the data sets should be directed to the corresponding author.
