# Supplementary material for: Sex differences in neurobehavior and the adult hippocampal neurogenic niche: influence of traumatic brain injury and CLIP antagonism
Source: Front Behav Neurosci. 2026 Feb 23;20:1768730. doi: 10.3389/fnbeh.2026.1768730 (PMC12968301; doi:10.3389/fnbeh.2026.1768730)
Supplement: Supplementary file 1 [file Table_1.docx]

Sex differences in neurobehavior and the adult hippocampal neurogenic niche: influence of traumatic brain injury and CLIP antagonism

Supplementary Material

# Supplementary Data

## Supplementary Figures


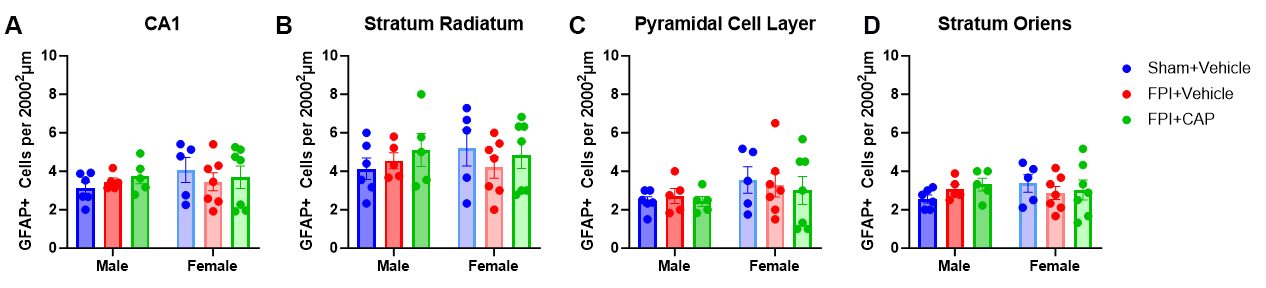


**Supplementary Figure 1.** **No changes to the GFAP+ astrocytes in CA1 following FPI or CAP treatment.** There were no significant differences identified in CA1 (**A**), or in any of the sublayers, including stratum radiatum (**B**), pyramidal cell layer (**C**), or stratum oriens (**D**). Data are represented as Mean ± SEM, n=6-8 per group.
